# Neighborhood-Level Food Insecurity and Colorectal Cancer Incidence in Texas: A Spatial Ecological Analysis

**DOI:** 10.21203/rs.3.rs-10502736/v1

**Published:** 2026-08-06

**Authors:** Ryan Ramphul, Craig Gundersen, Jooyeon Lee, Yanchen Liu, Yixiao Chen, Ruth Rechis, Lorna McNeill, Karen Basen-Engquist

**Affiliations:** The University of Texas Health Science Center at Houston; Baylor University; The University of Texas Health Science Center at Houston; The University of Texas Health Science Center at Houston; The University of Texas Health Science Center at Houston; The University of Texas MD Anderson Cancer Center; The University of Texas MD Anderson Cancer Center; The University of Texas MD Anderson Cancer Center

**Keywords:** CRC risk, Food insecurity, RUCA codes, Spatial model, Census tract

## Abstract

**Purpose:**

Colorectal cancer (CRC) remains a leading cause of cancer morbidity and mortality in the United States. Growing evidence suggests that neighborhood-level social drivers, including food insecurity and rurality, contribute to disparities in CRC burden, yet the joint effects of time-varying food insecurity and CRC remain understudied.

**Methods:**

We conducted a spatial ecological analysis of CRC incidence across 6,808 census tracts in Texas from 2017 to 2021. Tract-level CRC cases from the Texas Cancer Registry were linked with time-varying food insecurity estimates from Feeding America’s Map the Meal Gap and rural and urban classifications based on 2020 Rural–Urban Commuting Area (RUCA) codes. Sequential spatial Poisson models were used to assess associations between food insecurity, rurality and urbanicity, and CRC screening prevalence with CRC incidence.

**Results:**

Higher food insecurity was consistently associated with increased CRC incidence. In the unadjusted model, each one-percentage point increase in food insecurity corresponded to a 1.7% increase in CRC risk (RR = 1.017; 95% CrI, 1.015—1.019). When additional covariates were included, food insecurity was attenuated (RR range: 1.011—1.016). Compared with metropolitan areas, CRC risk was higher in micropolitan, small town, and rural tracts. Screening prevalence among older adults was negatively associated with CRC incidence.

**Conclusion:**

Food insecurity is a significant contextual factor that is positively associated with higher risk of CRC, independent of geographic settings and screening patterns. These findings highlight the structural disadvantage of food insecurity in shaping CRC burden in Texas and call attention to public health and social interventions to reduce CRC disparities.

## Introduction

Colorectal cancer (CRC) remains one of the leading causes of cancer-related morbidity and mortality in the United States, ranking third in cancer deaths for both men and women [1]. Although behavioral and genetic factors contribute to CRC risk, growing evidence highlights the role of social and structural conditions, including income, healthcare access, and neighborhood context, in shaping disparities in screening, diagnosis, and survival [2–4]. These conditions vary substantially across communities, suggesting that place-based factors may contribute to geographic inequalities in CRC.

Food insecurity, defined as limited or uncertain access to enough food for an active life, has been linked to poorer health outcomes and may adversely influence cancer prevention and treatment through delayed care, financial trade-offs, and nutritional deficits [5–9]. Despite this, few population-based studies have examined the relationship between food insecurity and CRC specifically, and even fewer have investigated how geographic context, particularly rurality, may modify this relationship. Rurality and urbanicity are particularly relevant in the context of CRC because rural communities consistently experience lower screening availability, longer travel distances to endoscopy services, and fewer healthcare resources, all of which contribute to elevated CRC incidence and later-stage diagnosis [10–13]. Prior research has identified broad geographic clustering of food insecurity or cancer outcomes [14–16], but no studies have jointly assessed food insecurity, rural–urban differences, and CRC risk using tract-level spatial methods across an entire state.

To address this gap, we analyzed Texas cancer registry data from 2017–2021 linked with census tract–level food insecurity estimates and rural–urban commuting classifications. Using Bayesian spatial Poisson models, we quantified how food insecurity and rurality independently and jointly relate to CRC risk.

## Methods

### Food Insecurity

Census tract-level food insecurity rate estimates in Texas from 2017 to 2021 were derived utilizing a methodological framework of Feeding America’s Map the Meal Gap (MMG), as described in Gundersen et al. (2014) [17]. Food insecurity estimates were expressed as percentages at the census tract level. Because census tract boundaries differ between the 2010 and 2020 definitions, we converted 2010 tract boundaries to the corresponding 2020 tracts using the comparability relationship records from the U.S. Census Bureau [18] before merging with the CRC data. Figure 2 displays the spatial distribution of food insecurity across Texas census tracts, averaged over the 2017–2021 period. Food insecurity showed a persistent geographic pattern across all five years, with the highest rates concentrated along the U.S.–Mexico border and throughout East Texas.

### Urbanicity and Rurality

The 2020 Rural–Urban Commuting Area (RUCA) codes (U.S. Department of Agriculture; USDA) [19] classifies U.S. census tracts on a scale from 1 to 10, with 1 being most urban and 10 being most rural. RUCA codes incorporate measures of population density, urbanization, and daily commuting patterns, providing a more nuanced measure than just population. More specifications of each RUCA code are displayed in Table 1. For interpretability and to align with common practices in health research using RUCA classifications [20, 21], we grouped codes 1–3 as metropolitan, codes 4–6 as micropolitan, codes 7–9 as small town, and code 10 as rural. This four-category grouping follows USDA-recommended RUCA aggregation schemes commonly used in health research [21].

### Colorectal Cancer Screening Prevalence

Estimates of CRC screening prevalence for people 50–75 years old is provided by 2023 CDC PLACES [22]. We used 2023 PLACES data because the estimates were generated from 2021 Behavioral Risk Factor Surveillance System, 2010 Census population, and 2015–2019 American Community Survey estimates which aligned best with our study period 2017–2021. Screening prevalence was included as a tract-level covariate to account for geographic variation in preventive care and early detection, which directly influences observed CRC incidence and mortality through adenoma removal and differential case ascertainment [23–25].

### Colorectal Cancer

**Figure 1:**
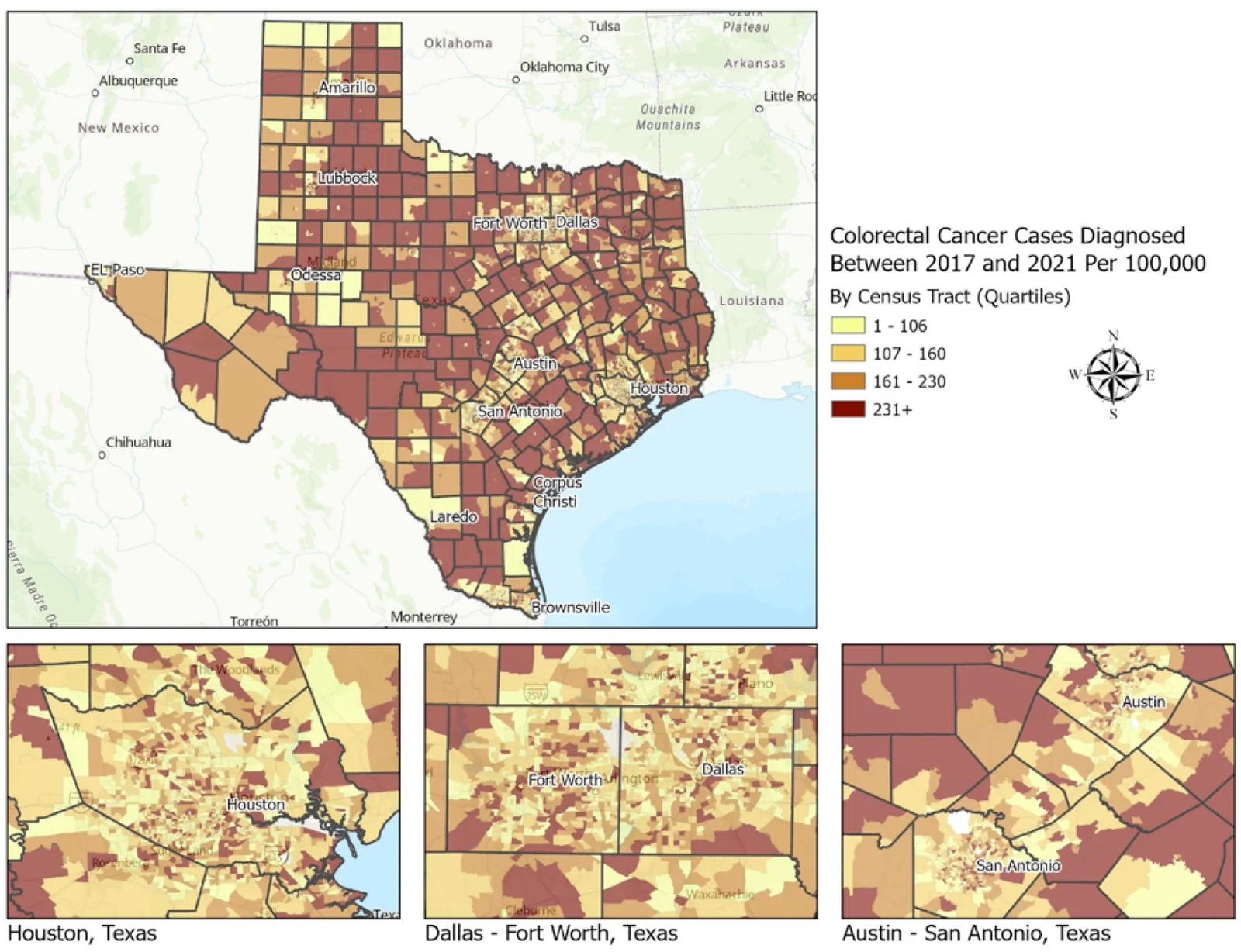
Colorectal Cancer Cases Diagnosed Between 2017 and 2021 Per 100,000 People, By Census Tract.

**Figure 2:**
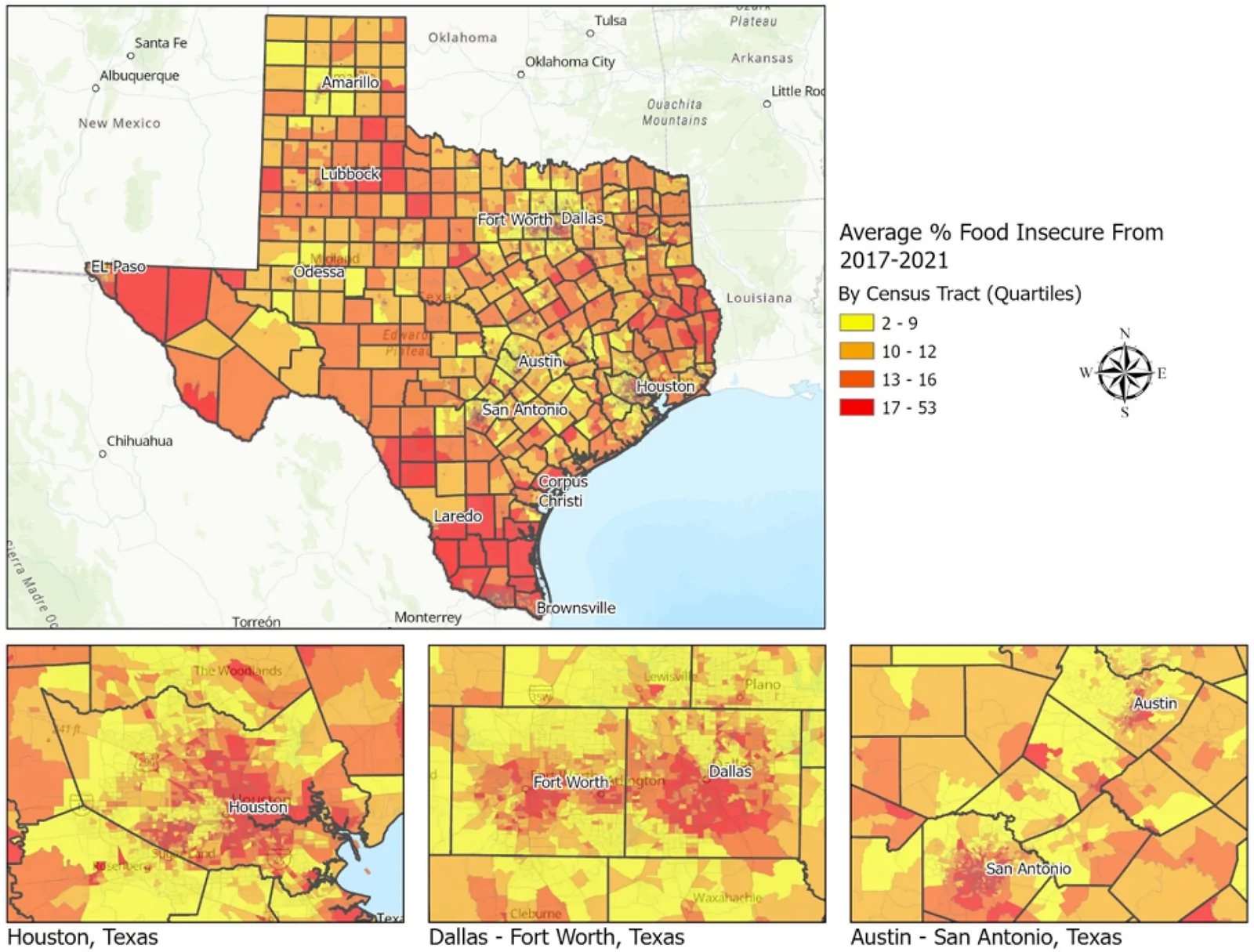
Average Food Insecurity Rates From 2017–2021, by Census Tract.

Colorectal cancer (CRC) patient data (C18–20 in ICD-10 codes) from the Texas Cancer Registry (TCR) [26] for cases diagnosed between 2017 and 2021 were included in our analyses. The TCR, one of the largest population-based cancer registries in the United States, monitors and reports the cancer burden in Texas. The dataset included patient addresses, demographic characteristics, and CRC diagnoses. Patient-level cases were aggregated to the census tract level for tract-level analyses. Figure 1 depicts CRC incidence per 100,000 persons between 2017 and 2021. Elevated rates were evident in rural and micropolitan regions, particularly the Texas Panhandle and East Texas, while, on average, lower CRC incidence was observed in major urban centers, with notable within-city variation across census tracts.

Table 2 summarizes CRC incidence rates per 100,000 persons in Texas from 2017 to 2021, overall and stratified by sex, age, and race/ethnicity, along with food insecurity distributions across aggregated USDA Rural-Urban Commuter Area (RUCA) codes. Each incidence rate is adjusted for the population at risk. Across the study period, CRC incidence varied by sex, age, and race/ethnicity. Incidence rates were higher among males than females and increased sharply with age, particularly among adults aged 55 years and older. Differences in CRC incidence were also observed across racial and ethnic groups, with higher crude incidence rates among White and Black or African American populations compared with other groups.

### Statistical Methods

All 6,896 Texas census tracts with a nonzero residential population were eligible for inclusion. However, we excluded tracts with fewer than 1,000 residents because these areas were predominantly nonresidential (e.g., airports, industrial zones, military zones) and if included, could generate unstable and unreliable estimates due to highly inflated incidence rates [27]. After exclusions, 6,808 out of 6,896 tracts remained for analysis. Across these tracts, the CRC incidence rate per 1000,000 persons ranged from 0 to 1,128.35 across study years (2017–2021), with an overall mean and standard deviation (SD) of total incidence per 100,000 people being 193 (SD = 110) cases. Given the low average number of cases and the count-based nature of the data, CRC incidence was modeled using Poisson regression. To account for spatial correlation among neighboring tracts, we implemented a Besag–York–Mollié 2 (BYM2) hierarchical spatial model [28]. This Bayesian framework decomposes variation into two components: (1) an unstructured random effect capturing random noise, and (2) a structured spatial effect capturing dependence among first order adjacent tracts. The model incorporated each tract’s expected number of CRC cases standardized by sex, age, and race/ethnicity and tract-level covariates representing food insecurity, rural–urban classification, and colorectal cancer screening prevalence among 50–75-year-old adults.

For the primary analysis, we fitted four spatial Poisson models to examine associations between census tract–level CRC cases and census tract-level food insecurity rates. Our unadjusted model A1 fitted a pooled 5-year model (2017–2021) that included food insecurity, a fixed effect for calendar year, along with a tract-level spatial random effect to adjust for temporal trends and persistent latent spatial variation in CRC risk. On top of model A1, we adjusted for rurality and urbanicity (model A2), colorectal cancer screening prevalence on adults aged 50–75 (model A3), and both (model A4). By using longitudinal data across the full 2017–2021 study period and explicitly modeling temporal trends with time-varying food insecurity, our approach leverages the full sample size to produce more stable tract-level estimates and narrower uncertainty intervals than single-year analyses, while avoiding bias from year-to-year variations.

The formulas for each of our models are as follows:

- Model A1. log (*RR*_*it*_) = log (*E*_*it*_) + *β*_0_ + *β*_1_*Food Insecurity*_*it*_ + *year*_*t*_ + *tract*_*i*_
- Model A2. log (*RR*_*it*_) = log (*E*_*it*_) + *β*_0_ + *β*_1_*Food Insecurity*_*it*_ + *β*_2_*RUCA category*_*i*_ + *year*_*t*_ + *tract*_*i*_
- Model A3. log (*RR*_*it*_) = log (*E*_*it*_) + *β*_0_ + *β*_1_*Food Insecurity*_*it*_ + *β*_2_*CRC screening*_*i*_ + *year*_*t*_ + *tract*_*i*_
- Model A4. log (*RR*_*it*_) = log (*E*_*it*_) + *β*_0_ + *β*_1_*Food Insecurity*_*it*_ + *β*_2_*RUCA category*_*i*_ + *β*_3_*screening*_*i*_ + *year*_*t*_ + *tract*_*i*_,

where tract is indexed by *i* = 1,…, 6,896 and year is indexed by *t* = 1,…, 5. *RR*_*it*_ denotes relative risk (RR) at tract *i* and year *t*, the offset term log (*E*_*it*_) is the expected number of CRC cases in tract *i* and year *t* internally standardized by sex-age-race/ethnicity, with *E*_*it*_ = ∑ _*j*_*N*_*ij*_*r*_*tj*_, where *j* is sex-age-race/ethnicity stratum, *N*_*ij*_ is time-invariant population, and *r*_*tj*_ is standardized rate (*r*_*tj*_ = ∑ _*i*_*y*_*itj*_/∑ _*i*_*N*_*ij*_) for tract *i* and stratum *j β*_0_ is an intercept, *year*_*t*_ represents fixed effects for calendar year, and *tract*_*i*_ is the spatial random effect modeled through spatial dependence as described in BYM2 model [28].

## Results

Table 3 presents the pooled model estimates describing associations between tract-level food insecurity and colorectal cancer (CRC) incidence across four sequential spatial Poisson models. Because food insecurity was the primary exposure of interest, results are presented first for food insecurity, followed by covariates included for adjustment (rurality and urbanicity and CRC screening prevalence).

Higher food insecurity was consistently associated with increased CRC risk across all models. In the unadjusted model (A1), each one–percentage point increase in food insecurity was associated with a 1.7% increase in CRC incidence (RR = 1.017; 95% CrI, 1.015–1.018). Adjustment for rurality and urbanicity (A2) minimally attenuated this association (RR = 1.016; 95% CrI, 1.014–1.018). Adjustment for CRC screening prevalence (A3) reduced the magnitude further (RR = 1.013; 95% CrI, 1.011–1.015), and the fully adjusted model including both rurality and screening (A4) yielded an RR of 1.011 (95% CrI, 1.009–1.013). These associations indicate that tract-level food insecurity remains a significant contextual correlation of CRC incidence independent of geographic setting and screening patterns.

Rurality and urbanicity and CRC screening prevalence were included in the models to account for geographic variation in healthcare access and cancer detection, and their associations with CRC risk were also evaluated. Compared with metropolitan tracts, micropolitan areas had 14–16% higher CRC risk, small town areas had 19–21% higher CRC risk, and rural areas had 12–14% higher risk across models A1-A4. CRC screening prevalence was inversely associated with CRC incidence (RR range, 0.996–0.994), indicating that tracts with higher screening prevalence had lower observed CRC incidence after accounting for food insecurity and rurality and urbanicity. Together, these covariate associations help isolate the independent relationship between food insecurity and CRC incidence.

## Discussion

This study examined census tract-level food insecurity rates in relation to colorectal cancer (CRC) incidence across Texas census tracts from 2017 to 2021. Using a spatial Bayesian Poisson model with time-varying food insecurity and pooled CRC incidence, we found that higher food insecurity was consistently associated with higher CRC incidence across all models. Even after adjusting for rurality and urbanicity and CRC screening prevalence, food insecurity remained a significant predictor of elevated CRC risk. These findings suggest that food insecurity captures meaningful contextual disadvantage relevant to CRC burden in Texas.

Rurality and urbanicity and screening prevalence behaved as expected for contextual covariates. Rural, small town, and micropolitan tracts demonstrated higher CRC incidence than metropolitan areas, consistent with persistent geographic disparities in access to preventive services, specialty care availability, and broader health system capacity [29–31]. Screening prevalence was inversely associated with CRC incidence after adjustment, reflecting the well-documented protection conferred by routine screening with established prevention benefits of screening. A study also marked the impact of colonoscopy screening in reducing the CRC incidence and mortality due to the early detection and removal of adenomas [32]. These covariates refine, but do not diminish, the association between food insecurity and CRC.

Food insecurity likely functions as an area-level indicator of structural hardship, including concentrated poverty, resource scarcity, and constrained access to health-supporting environments. Although our ecological design does not permit inference about individual pathways, the spatial clustering of food insecurity aligns with the geographic distribution of CRC burden, reinforcing evidence that neighborhood disadvantage shapes chronic disease risk.

A key strength of this study is the integration of statewide, census tract–level cancer registry data with longitudinal, time-varying estimates of food insecurity and contextual measures of rurality and colorectal cancer screening. This design allows for the examination of persistent geographic disparities in CRC incidence across Texas while accounting for both structural and health system–related factors. The use of a Bayesian spatial modeling framework, including the BYM2 specification, supports this analysis by appropriately accounting for spatial autocorrelation and stabilizing estimates in sparsely populated areas.

These findings underscore opportunities to integrate food insecurity interventions into cancer prevention and control strategies. A key tool that can be used is the Supplemental Nutrition Assistance Program (SNAP). This program leads to reductions in food insecurity [33] so encouraging participation is one intervention that can be pursued. Increasing participation is especially important for older Americans who have lower participation rates among eligible households (55% in comparison to non-senior adults, 95% [34]) and higher probabilities of CRC. Integrating food insecurity screening into primary care, oncology, and community-based health settings; and co-locating CRC screening services with food distribution sites or community health centers may help reduce barriers to preventive care and early detection. These strategies highlight the potential for cross-sector partnerships between public health, health care, and social services to reduce CRC.

Several limitations warrant consideration. As an ecological study, the findings describe associations at the community level and cannot be interpreted as individual-level causal effects. Unmeasured tract-level factors, including environmental exposures, may contribute to residual spatial variation. Screening prevalence was obtained from modeled CDC PLACES data, which may not fully capture within-tract heterogeneity or actual screening availability or modality; thus, it should be interpreted as a contextual approximation.

Despite these limitations, this study demonstrates that neighborhood food insecurity is associated with CRC incidence, independent of geographic setting and screening patterns. Addressing food insecurity and related social needs at the community level may offer a promising pathway to reduce CRC disparities and improve population health across Texas.

## Data acknowledgement statement

Texas cancer data have been provided by TCR, the Cancer Epidemiology and Surveillance Branch, Texas Department of State Health Services, 1100 West 49th Street, Austin, TX 78756. Data from TCR is supported by the following: Cooperative Agreement #1NU58DP007140 from the Centers for Disease Control and Prevention (CDC), Contract #75N91021D00011 from the National Cancer Institute’s Surveillance, Epidemiology, and End Results (SEER) Program, and the Cancer Prevention and Research Institute of Texas (CPRIT).

## Figures and Tables

**Table 1:** Rural–Urban Commuting Area (RUCA) classification used in this study.

RUCA codes	RUCA category	Description
1	Metropolitan	Metropolitan core areas
2		Metropolitan areas with high commuting
3		Metropolitan areas with low commuting
4	Micropolitan	Micropolitan core areas
5		Micropolitan areas with high commuting
6		Micropolitan areas with low commuting
7	Small town	Small town core areas
8		Small town areas with high commuting
9		Small town areas with low commuting
10	Rural	Rural areas with minimal commuting

Note: RUCA codes are whole numbers (1–10) defined based on population density, urbanization, and commuting patterns. Lower values indicate more urbanized areas.

**Table 2:** Summary statistics of colorectal cancer (CRC) incidence and census tract–level characteristics across the full 2017–2021 study period.

Newly Diagnosed CRC Cases 2017–2021					
	Overall; N = 6,808	Rurality-Urbanicity			
		Metropolitan (RUCA 1–3); N = 5,779	Micropolitan (RUCA 4–6); N = 477	Small town (RUCA 7–9); N = 158	Rural (RUCA 10); N = 394
**Population characteristics**					
**Overall (per 100,000; Mean (SD))**	193 (110)	178 (98.1)	260 (120)	296 (138)	295 (146)
**Sex (per 100,000; Mean (SD))**					
Female	172 (126)	159 (115)	225 (134)	281 (177)	255 (178)
Male	217 (149)	200 (134)	299 (166)	322 (194)	340 (202)
**Age (per 100,000; Mean (SD))**					
Under 5 years	0 (0)	0 (0)	0 (0)	0 (0)	0 (0)
5 to 9 years	1.02 (33.3)	1.10 (35.7)	0.214 (4.67)	1.21 (15.2)	0.869 (17.3)
10 to 14 years	2.89 (45.0)	2.15 (34.2)	7.71 (70.1)	9.80 (90.8)	5.28 (92.5)
15 to 17 years	4.16 (74.4)	3.39 (68.4)	2.59 (56.5)	6.88 (86.5)	16.2 (142)
18 and 19 years	14.6 (323)	15.5 (344)	15.4 (215)	5.36 (67.4)	4.03 (80.0)
20 to 24 years	13.2 (152)	13.9 (161)	10.0 (99.4)	9.32 (60.3)	7.11 (70.6)
25 to 29 years	18.9 (146)	18.6 (141)	26.9 (214)	24.0 (160)	11.8 (85.1)
30 to 34 years	36.6 (190)	35.8 (169)	38.0 (172)	30.1 (147)	49.9 (405)
35 to 44 years	83.7 (190)	80.1 (182)	108 (241)	113 (262)	95.5 (194)
45 to 54 years	270 (397)	261 (358)	308 (336)	318 (409)	332 (800)
55 to 64 years	457 (651)	439 (671)	574 (488)	612 (525)	528 (530)
65 to 74 years	754 (1,030)	740 (1,070)	843 (702)	846 (773)	819 (628)
75 to 84 years	1,190 (3,450)	1,140 (25,70)	1,700 (9,290)	1,460 (1,630)	1,250 (1,650)
85 years and over	1,530 (4,750)	1,450 (4,740)	1,940 (5,380)	2,190 (5,030)	1,910 (3,830)
**Race/Ethnicity (per 100,000, Mean (SD))**					
Hispanic	140 (501)	127 (272)	182 (442)	403 (2690)	167 (318)
Non-Hispanic Black or African American	346 (2,600)	339 (2,690)	396 (1,980)	547 (2,720)	314 (1,690)
Non-Hispanic White	395 (1,650)	395 (1,780)	389 (693)	405 (268)	399 (321)
Non-Hispanic Asian	212 (2,080)	205 (1,660)	358 (4,890)	145 (1,410)	177 (2,120)
Non-Hispanic Natives	149 (2,610)	105 (1,910)	94.9 (1,560)	692 (7,980)	636 (5,960)
Non-Hispanic Others	20.1 (630)	22.2 (681)	18.7 (225)	0 (0)	0 (0)
**Tract-level characteristics**					
**CRC screening prevalence on adults 50–75 years old (mean (SD))**	58.3 (7.58)	58.0 (7.74)	58.8 (6.61)	59.3 (6.08)	60.8 (6.24)

CRC incidence rates are crude and pooled across the full 2017–2021 study period. Values are descriptive and not intended for statistical inference. Food insecurity varies annually, whereas rurality and urbanicity and CRC screening prevalence were treated as time-invariant census tract–level covariates in regression analyses. CRC screening prevalence estimates were obtained from CDC PLACES (2023).

**Table 3:** Results of association between CRC cancer risk and food insecurity among census tracts with population ≥1,000. Results are presented for unadjusted or adjusted for either 1) rurality and urbanicity, 2) CRC screening, or 3) rurality and urbanicity and CRC screening.

Food insecurity (%)	Unadjusted (Model A1)	Model including RUCA (Model A2)	Model including CRC screening (Model A3)	Model including RUCA & CRC screening (Model A4)
	1.017 (1.015 1.019) *	1.016 (1.014 1.018) *	1.013 (1.011 1.015) *	1.011 (1.009 1.013) *
**Rurality and Urbanicity**				
Metropolitan(ref)				
Micropolitan		1.145 (1.104 1.188) *		1.157 (1.115 1.2) *
Small town		1.114 (1.069 1.161) *		1.135 (1.089 1.182) *
Rural		1.194 (1.124 1.27) *		1.211 (1.139 1.287) *
**CRC screening prevalence on adults 50–75 years old**			0.996 (0.994 0.997) *	0.994 (0.993 0.996) *

* Indicates 95% Bayesian credible interval (CrI) for odds ratio that does not include the null value of 1, indicating strong evidence that the association is different from the null.

## Data Availability

The datasets generated and/or analyzed during the current study are not publicly available due to data use restrictions and the need to protect individual privacy. Texas cancer data have been provided by TCR, the Cancer Epidemiology and Surveillance Branch, Texas Department of State Health Services, 1100 West 49th Street, Austin, TX 78756. Data from TCR is supported by the following: Cooperative Agreement #1NU58DP007140 from the Centers for Disease Control and Prevention (CDC), Contract #75N91021D00011 from the National Cancer Institute’s Surveillance, Epidemiology, and End Results (SEER) Program, and the Cancer Prevention and Research Institute of Texas (CPRIT).
